# Epigenetics as an answer to Darwin’s “special difficulty,” Part 2: natural selection of metastable epialleles in honeybee castes

**DOI:** 10.3389/fgene.2015.00060

**Published:** 2015-02-24

**Authors:** Douglas M. Ruden, Pablo E. Cingolani, Arko Sen, Wen Qu, Luan Wang, Marie-Claude Senut, Mark D. Garfinkel, Vincent E. Sollars, Xiangyi Lu

**Affiliations:** ^1^Department of Obstetrics and Gynecology, C. S. Mott Center for Human Growth and Development and Center for Urban Responses to Environmental Stressors, Institute of Environmental Health Sciences, Wayne State UniversityDetroit, MI, USA; ^2^School of Computer Science and Genome Quebec Innovation Centre, McGill UniversityMontreal, QC, Canada; ^3^Department of Pharmacology, Wayne State UniversityDetroit, MI, USA; ^4^Institute of Environmental Health Sciences, Wayne State UniversityDetroit, MI, USA; ^5^Department of Biological Sciences, University of Alabama in HuntsvilleHuntsville, AL, USA; ^6^Department of Biochemistry and Microbiology, Joan C. Edwards School of Medicine, Marshall UniversityHuntington, WV, USA

**Keywords:** epigenetics, evolution, genomics, developmental plasticity, eusociality, group selection

## Abstract

In a recent perspective in this journal, Herb (2014) discussed how epigenetics is a possible mechanism to circumvent Charles Darwin’s “special difficulty” in using natural selection to explain the existence of the sterile-fertile dimorphism in eusocial insects. Darwin’s classic book “On the Origin of Species by Means of Natural Selection” explains how natural selection of the fittest individuals in a population can allow a species to adapt to a novel or changing environment. However, in bees and other eusocial insects, such as ants and termites, there exist two or more castes of genetically similar females, from fertile queens to multiple sub-castes of sterile workers, with vastly different phenotypes, lifespans, and behaviors. This necessitates the selection of groups (or kin) rather than individuals in the evolution of honeybee hives, but group and kin selection theories of evolution are controversial and mechanistically uncertain. Also, group selection would seem to be prohibitively inefficient because the effective population size of a colony is reduced from thousands to a single breeding queen. In this follow-up perspective, we elaborate on possible mechanisms for how a combination of both epigenetics, specifically, the selection of metastable epialleles, and genetics, the selection of mutations generated by the selected metastable epialleles, allows for a combined means for selection amongst the fertile members of a species to increase colony fitness. This “intra-caste evolution” hypothesis is a variation of the epigenetic directed genetic error hypothesis, which proposes that selected metastable epialleles increase genetic variability by directing mutations specifically to the epialleles. Natural selection of random metastable epialleles followed by a second round of natural selection of random mutations generated by the metastable epialleles would allow a way around the small effective population size of eusocial insects.

## DARWIN’S “SPECIAL PROBLEM”

Eusocial (Greek *eu*: “good/real” + “social”) insects include the Hymenoptera (ants, bees, and wasps) and the Isoptera (termites). In honeybees, which is the focus of this perspective, a hive has caste differences; the diploid queen and the haploid drones are the sole reproducers, while the nurses, soldiers, guards, and foragers are “sub-castes” or “task groups” of the worker caste of sterile females that work together to benefit the group as a whole (Free, 1987). However, as pointed out by Herb (2014) in a previous perspective in this journal, having sterile females in a colony is a potentially fatal flaw in Darwin’s theory of natural selection, which states that the fittest individuals pass their traits (i.e., genes) to the next generation. Darwin (1859) referred to sterile workers in insect communities as, “… one special difficulty, which at first appeared insuperable, and actually fatal to the whole theory.” Darwin (1871) later proposed a way around this “special difficulty” by proposing a “group selection” model for evolution of altruistic behaviors in eusocial insects. Darwin argued that “group selection” can occur when the benefits of altruism between castes are greater than the individual benefits of selfishness (egotism) within a subpopulation. Hamilton (1964), the great population geneticist Hamilton formalized the idea of group selection in a mathematical model, rb > c, where b represents the benefit to the recipient of altruism, c the cost to the altruist, and r their degree of relatedness. Kin selection takes into account the genetic relatedness of individuals in a group, was a further refinement of the group selection theory. In kin selection, rb_k_ + b_e_ > c, in which b_k_ is the altruistic benefit to kin and b_e_ is the altruistic benefit accruing to the group as a whole (Wilson and Wilson, 2007). However, group- and kin- selection models are mathematically complex and remain controversial amongst many evolutionary theorists, such Dawkins (1976) and Nowak et al. (2010), who argue that group selection is unlikely because, among many reasons, selfishness (i.e., “selfish genes” – a phrase Dawkin’s coined) would always predominate over altruism.

Here, we further elaborate on Herb’s (2014) thesis that epigenetics might be a way around Darwin’s “special difficulty.” We argue that epigenetic inheritance systems (EISs) can allow rapid evolution of traits specific for sterile workers and fertile queens. Epigenetics does not involve changes in the DNA sequence, but rather covalent, yet reversible, changes to the DNA in the form of 5-methylcytosine (5mC). EISs should work fine for short-term evolutionary changes. However, natural selection of DNA sequence variants would still be needed for long-term evolutionary changes. Histone modifications, long non-coding RNAs, prions, and other types of EISs will not be discussed in detail, but rather we will focus on 5mC, since 5mC represents a reversible change to the genome that can be modified by the environment (Chia et al., 2011). Heritable changes in 5mC, such as occurs in imprinted genes in mammals, are also called metastable epialleles (Rakyan et al., 2002; Dolinoy et al., 2007). The most important aspect of DNA methylation in the hypothesis presented in this paper is that, unlike histone modifications, 5mC is mutagenic and can lead to permanent changes to the DNA. Specifically, 5mC can undergo spontaneous deamination, which converts 5mC to T (Coulondre et al., 1978; Duncan and Miller, 1980). A hypothesis for how natural selection of metastable epialleles can lead to DNA mutations that permanently stabilize the epialleles into real alleles, the epigenetic directed genetic error (EDGE) hypothesis, is presented in the last section of this perspective.

The inspiration for many of the ideas in this perspective is a chapter in Jablonka and Lamb’s (2005) excellent book “Evolution in Four Dimensions” on EISs. They created an imaginary planet named Jaynus where the variety of organisms all had exactly the same genome sequences, yet had many different phenotypes. They wrote:

Jaynus organisms have a genetic system that is based on DNA, and replication transcription, and translation are much the same as on Earth. However, there is one very extraordinary thing about the DNA of Jaynus creatures – every organism has exactly the same DNA sequences. From the simplest organism, a tiny unicellular creature, to the enormous fanlike colonial worms, the DNA is identical. Their genomes are large and complex, but no organism deviates from the universal standard sequences because there are cellular systems that check DNA and destroy any cell suspected of carrying a mutation.

In this perspective, we describe how intra-caste evolution is, in many respects, similar to how evolution proceeds on the mythical planet of Jaynus.

## MECHANISMS OF CASTE DETERMINATION AND EPIGENETIC MODIFICATION IN HONEYBEES

*At the extreme superorganismic phase, the level of selection becomes the genome of the queen and the sperm she stores, and the workers can be viewed as robotic extensions of her phenotype (Wilson and Nowak, 2014*).

As beautifully described in the above quote, eusocial insects are even more extreme in some respects than the mythical organisms on Jaynus because they have evolved to a “superorganismic” stage in which the queen is the reproductive organ(ism) and the workers are the “robotic extensions” or the somatic cells of the superorganism. Kennedy et al. (2014) called the formation of eusocial insect colonies and super colonies “a new major transition in evolution.” We propose that the intra-caste evolutionary process in honeybees might share epigenetic mechanisms with those proposed on Jaynus. Darwin (1859) evolution utilizes the concepts of “survival of the fittest” in a population and “natural selection” of genetic variation to eventually form new species. Darwin, of course, did not know about either genetic or epigenetic variation, for his work pre-dated Gregor Mendel’s discoveries (or, more accurately, rediscovery in the 20th century (Meneses Hoyos, 1960; Fairbanks and Rytting, 2001), but the modern interpretation of “natural selection” is selection of genetic variation. We propose that “intra-caste evolution” is a type of micro-evolution, which is small-scale evolution within a population, and refers to survival of the fittest members of a caste. We propose here that “intra-caste evolution” is initially based on natural selection of metastable epialleles of the most-fit caste (i.e., queen and worker) and sub-caste members (i.e., nurse, soldier, guard, and forager). As in the mythical Jaynus example, genetic selection probably cannot be the primary mechanism for selecting the fittest worker bee, since most worker bees cannot breed (however, see below). For example, the most efficient forager sub-caste of workers cannot be selected for by direct genetic selection, since workers, in most situations, are sterile females. However, a hive with more efficient foragers can be produced by group selection of metastabile epialleles that produce an increased foraging efficiency. One phenotype that worker bees have evolved to increase foraging efficiency are the pollen baskets on the hind legs, which are present on workers but not on queens. A possible mechanism for the evolution of pollen baskets is presented later in this perspective.

If the “intra-caste evolution” hypothesis of honeybee castes is not mediated primarily by genetic means, then how are the desirable phenotypes, such as efficiency in foragers, transmitted to the next generation? We propose that, first, queens undergo a great deal of stress (i.e., malnutrition) when there is not an adequate amount of foraging being performed by the workers. The stress, in a mechanism that we present in a later section, leads to an activation of random stress-induced metastable epialleles, some of which increase the food-carrying capacity of pollen baskets. Second, the metastable epialleles which improve the fitness of the colony, such as those that serendipitously alter pollen baskets in workers in a manner that increases storage capacity, are selected over several generations by group selection. Third, random mutations can potentially be directed to the selected metastable epialleles by the EDGE mechanism, described in the final section of this perspective. The main reason for the need for the EDGE hypothesis is, we believe, because the “normal” background mutation and group selection processes are not adequate when the effective population size of a species is too low, as it arguably is in eusocial insects (i.e., only the queen breeds). The EDGE hypothesis provides an additional mechanism to increase the mutation rate of specific genes required to ensure the survival of the colony.

## CHEMICAL MEANS OF EPIGENETIC MODIFICATIONS IN HONEYBEE CASTES

In addition to the selection of the most-fit caste and sub-caste members in each generation by group selection, honeybees have evolved to produce royal jelly to alter the epigenetic and developmental machinery of their offspring. The active ingredients of royal jelly include a fatty acid, (E)-10-hydroxy-2-decenoic acid (10HDA), which accounts for up to 5% of royal jelly. The fatty acid 10HDA, interestingly, is an epigenetic modifier molecule with a histone deacetylase inhibitor (HDACi) activity (Spannhoff et al., 2011). HDACs remove acetyl groups from histones, which are present in actively transcribed genes to open up the chromatin, presumably by repellent ionic charges pushing the nucleosomes apart (Jenuwein and Allis, 2001). HDACi’s inhibit the deacetylation of histones, which would lead to the acetyl groups remaining on histones, and therefore transcriptional activity would be high in the “queen-specific genes” of larvae fed royal jelly. Another component of royal jelly is the protein royalactin, which increases body size and ovary development in queens (Kamakura, 2011). The mechanism of action of royalactin is thought to be multifold: activation of mitogen-activated protein kinase (MAPK), which decreases developmental time, activation of p70 S6 kinase, which increases body size, and increasing juvenile hormone production, which is an essential hormone for ovary development (Kamakura, 2011). Interestingly, the same paper also showed that royalactin dramatically increases body size and ovary development when fed to the fruit fly, *Drosophila melanogaster* (Kamakura, 2011).

Based on the fact that royalactin has similar effects on the solitary fruit fly as on eusocial bees, we propose a theoretical epigenetic mechanism for how the queen’s dependence on royal jelly for ovary development evolved. In our model, bees originally were solitary, like fruit flies, and every female fended for herself in terms of feeding and reproduction. However, when the food is in short supply, the absence of nutrients would lead to a reduction of reproductive fitness and a diminution in ovary development. Consequently, the population reaches a bottleneck when the food runs low, and only those few individuals that have sufficient nutrition survive. If the few survivors evolved the capacity to feed some of their offspring, which would be one of the first steps in eusocial evolution, then when the food runs too low, they can feed adequately only some of their offspring and leave the other offspring malnourished. The female offspring that are fed would develop ovaries, whereas the female offspring that were not sufficiently fed would develop atrophied ovaries and would be sterile. A decrease in reproductive fitness is a universal character of most animals during starvation (Carey et al., 2008). However, and this is key, both fertile and sterile offspring are produced by the same mother, in a manner that is dependent on how much food or the quality of food they were fed. If the sterile offspring provided a selective-advantage to the group as a whole, then those mothers that produced both fertile and sterile offspring would have a selective advantage over those mothers that produced only fertile offspring. After millions of years of fine-tuning this process, the honeybee sterile-fertile dimorphism could have theoretically evolved by group selection.

As pointed out by an anonymous reviewer, there are at least three potential problems with our hypothesis on how honeybees evolved to produce royal jelly. First, the feeding behavior would need to be developed when food was scarce. Second, the sterile-fertile dimorphism would have to be maintained even though food became abundant again. Third, altruism would have to be developed when the sterile-fertile dimorphism emerged. It is hard to argue around these criticisms for a solitary insect such as *Drosophila*, and that might be why *Drosophila* and other solitary insects never evolved a sterile-fertile dimorphism. However, as suggested by Wilson and Nowak (2014), perhaps a way to circumvent all of these problems is the fact that the first step in eusocial evolution is probably the ability to form nests or colonies. This would allow the development of the dichotomy in bees, wasps, and ants of being a forager or staying in the nest to lay eggs. Since foraging is dangerous and taxing, if the workers are bringing the proto-queen pollen and nectar, then she is less inclined to forage for it. Once the proto-queen evolved the ability to produce royal jelly, then she would become the only fertile member of the colony – all of the workers could be chemically sterilized by withholding royal jelly. The development of altruism, in this case, could be an emergent property of the sterile-fertile dimorphism. As discussed further in a later section, there are many examples of emergent behavior in eusocial insects (Johnson, 2001), and we argue that altruism of sterile workers could be one of them.

In addition to royal jelly, honeybees have evolved an arsenal of other chemical weapons that subvert developmental and behavioral processes in the workers. For example, after about 8 days post-emergence, the nurse bees who take care of the eggs will transition into foragers, and foraging is more metabolically taxing because it requires the filling of baskets on the hind legs to transport the pollen (Free, 1987). However, the behavioral transition from nurse to forager depends on the needs of the hive, and individuals in the hive transmit these needs by both direct contact and by pheromones. The transition among sub-caste members in honeybees, but not some ant species that have physically different worker castes (Wilson and Nowak, 2014), is purely behavioral because all honeybee workers have pollen baskets despite the fact that only foragers use them – pollen baskets do not develop *de novo* in the nurse when she transforms into a worker.

Queen mandibular pheromone (QMP) is emitted by the queen to recruit nurses to her and to suppress ovary growth (Free, 1987). The larvae emit brood pheromone (BP) to stimulate nurse bees to feed and care for them (i.e., the brood). BP affects the nurses and foragers in different manners: it stimulates nurses to care for the brood and to delay their transition into foragers, while it stimulates foragers to collect nutrient-rich pollen to feed the brood (Slessor et al., 2005). Foragers, in turn, emit ethyl oleate (EO) to suppress nurse honeybees from foraging (Leoncini et al., 2004). Isoamyl acetate, which has a similar odor to the banana and pear, was found in Boch et al. (1962) to be an active component in the sting pheromone of the honeybee which is presumably released by honeybee guards when a hive is disturbed. It is through such chemical (i.e., environmental) signals that the honeybees are able to epigenetically maintain the caste structure in a manner that circumvents, in most aspects, the need for the selection of genetic variation.

## THE POTENTIAL ROLE OF NATURAL SELECTION OF GENETIC AND EPIGENETIC VARIATION IN DRIVING THE EVOLUTION OF CASTE SYSTEMS

As mentioned earlier, an important consideration regarding genetic-stabilization of the sterile-fertile dimorphism, is that the sterile workers can, in rare cases, develop ovaries. Removal of a queen can cause some workers to develop ovaries, in part because they are no longer exposed regularly to QMP (Herb et al., 2012, 2013). Also, nurses and foragers can revert back-and-forth rapidly in either direction in a manner that is dependent on the needs of the hive (Amdam et al., 2005). We believe that the occasional reversion of a sterile worker to a reproductive female is a critical mechanism for transmitting both metastable epialleles and genetic variation that is required for the worker caste. According to the EDGE hypothesis, genetic variation that is induced by the metastable epialleles, can be selected to increase worker specialization in the next generation. The purpose of the metastable epialleles, in the EDGE hypothesis, are not to circumvent the need for genetic variation, but rather to increase genetic variation in precisely the genes that need to be adapted for the organism, or superorganism in the case of eusocial insects, to survive the novel environment.

The selection of metastable epialleles in *Drosophila* is well-established in our laboratory (Ruden et al., 2003, 2008, 2009; Sollars et al., 2003; Ruden and Lu, 2008) and in other laboratories (Carrera et al., 1998; Ruden and Lu, 2008; Tariq et al., 2009; Gangaraju et al., 2010; Valtonen et al., 2012; Branco and Lemos, 2014; Le Thomas et al., 2014; Nystrand and Dowling, 2014; Somer and Thummel, 2014; Stern et al., 2014; Wei et al., 2015). We showed, for instance, that stress, or the inactivation of the chaperone protein Hsp90, can activate a metastable epiallele of the *Kruepple*^Incomplete facets-1^ (*Kr*^If-1^) allele, which causes ectopic large bristle outgrowths (ELBOs) to protrude from the eyes (Sollars et al., 2003). We indicate the metastable epiallele with the nomenclature [*Kr*^If-1^]^∗^ and showed that the metastable epiallele can be transmitted through both the male and female germlines for tens or even hundreds of generations (Ruden et al., 2003, 2008). What makes a metastable epiallele an example of an epigenetic variant rather than a genetic variant is the fact that a metastable epiallele, such as [*Kr*^If-1^]^∗^, can be reverted back to the original allele, in this case *Kr*^If-1^, in just one or two generations by negative selection (Sollars et al., 2003). Since *Drosophila* has very little DNA methylation, the metastable epialleles in *Drosophila* are probably not the result of differential DNA methylation. However, Gangaraju et al. (2010) presented evidence that the [*Kr*^If-1^]^∗^ metastable epiallele requires Piwi and Pi RNAs, which are small non-coding RNAs in the germline and function similarly to siRNAs and mi-RNA (Ruden, 2011; Grentzinger et al., 2012). We are still actively trying to determine the exact nature of the [*Kr*^If-1^]^∗^ metastable epiallele and how it is transmitted through both the male and female germlines. As discussed later, we believe that *Drosophila*, and more generally most or all Dipterans (flies) and Coleopterans (beetles), lost DNA methylation because the presence of 5mC would slow down the syncytial blastoderm mitotic cycles, which at ∼8 min are the fastest in the animal kingdom (Ruden and Jackle, 1995).

There is no direct laboratory evidence that selection of metastable epialleles occurs in eusocial insects, such as honeybees. However, there are at least three indirect indications that metastable epialleles that utilize differential DNA methylation occur in eusocial insects. First, Herb et al. (2012, 2013) showed that reverting foragers back to nurses reestablished the nurse-pattern of DNA methylation. This was the first evidence of reversible epigenetic changes associated with behavior. Second, Hunt et al. (2010) found that worker-biased proteins exhibited slower evolutionary rates than queen biased proteins or non-biased proteins. This is consistent with the idea that metastable epialleles must be transmitted through the germline, and the queen and fertile workers are the only females that produce eggs. Finally, as described in the next section, the bimodal distributions of CG content and/or DNA methylation in most insect genes suggests a role for differential DNA methylation and the existence of metastable epialleles in most insects.

## MECHANISMS OF EPIGENETIC MODIFICATION IN HONEYBEES

**FIGURE 1:**
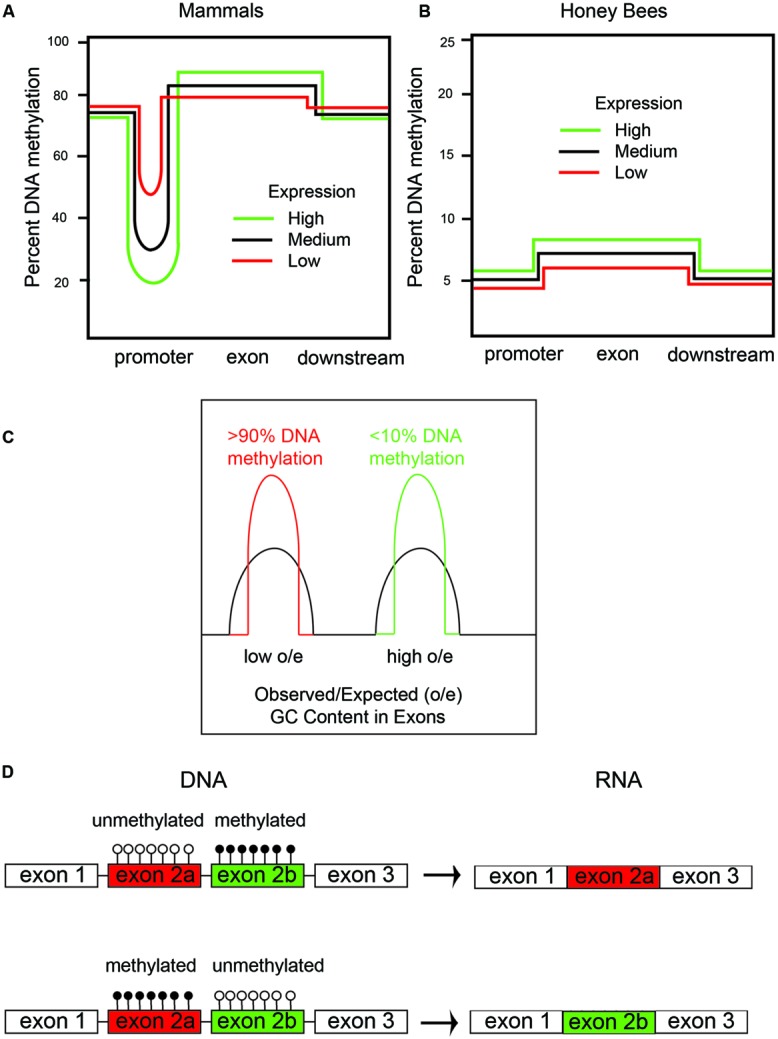
**DNA methylation in honeybees correlates with gene expression and alternative splicing. (A)** There are two types of DNA methylation in mammals: (1) promoter DNA methylation, which inversely correlates with mRNA expression; and (2) exon DNA methylation, which positively correlates with mRNA expression. **(B)** Honeybees predominantly have DNA methylation in exons, which, like in mammals, positively correlates with gene expression. **(C)** There are two types of genes in honeybees: (1) housekeeping genes with low observed/expected (o/e) CG content and high amounts of DNA methylation, and (2) caste-specific and developmental regulatory genes with a high o/e CG content and low amounts of DNA methylation. We have shown that the DNA methylation is at both CpG and CHH sites – CpG methylation primarily in exons and CHH methylation primarily in introns. **(D)** DNA methylation of cassette exons leads to their exclusion by alternative splicing in honeybees.

How might an EIS in honeybees and other organisms evolve? In order to understand this, it is necessary to describe the patterns of DNA methylation in mammals and honeybees (**Figures 1A,B**). In mammals, ∼60% of genes have so-called CpG islands in the promoter regions and 5′ regions, which are defined as regions of higher than average CG content. DNA methylation of CpG islands in mammals occurs primarily at CpG sites in somatic cells but often at CHH (where H = C, A or T) sites in stem cells (reviewed in Patil et al., 2014). The degree of CpG island methylation is inversely proportional to gene expression for most genes; i.e., highly expressed genes have little CpG island DNA methylation, whereas, low-expressed genes have large amounts of CpG island DNA methylation (**Figure 1A**). Two mechanisms that CpG island DNA methylation in mammals are thought to function to reduce gene expression are by inhibiting binding of some transcriptional activation factors, such as AP1, which binds to GC-rich consensus sequences, and by increasing the binding of transcriptional inhibitory factors, such as MeCP2, which recruits HDACs to inhibit transcription (reviewed in Jones, 2012).

The CpG island DNA methylation story is the most-well-known aspect of epigenetic regulation of transcription in mammals. However, several studies have shown that gene-body DNA methylation also occurs in a manner that is mostly proportional to gene expression in both mammals and insects (Konu and Li, 2002; reviewed in Jones, 2012). In other words, highly expressed genes have the most gene-body DNA methylation, and this DNA methylation is mostly restricted to exon sequences (**Figure 1B**), but this is partly because exons, since they encode proteins, are CG rich compared to intronic and intergenic regions, which do not encode proteins. DNA methylation in mammals also occurs at repeat sequences, such as ALUs, SINES, LINES, and retroviruses, and this has been shown to prevent expression and, thereby, retro-transposition of the retroviruses to new genomic regions (Jones, 2012).

Interestingly, DNA methylation in honeybees occurs primarily in gene bodies, particularly in exons (**Figure 1B**). However, in contrast to mammals, CpG islands are not apparent in the promoters of honeybee genes (i.e., there are very few genes with enriched CG-content in the promoter regions). Additionally, in the honeybee, little or no DNA methylation occurs in repeat or intergenic sequences (Lyko et al., 2010; Zemach et al., 2010; Chen et al., 2011). Therefore, in honeybees, DNA methylation is not thought to epigenetically regulate expression of genes by controlling transcription factor binding to promoter regions, but rather is a consequence of gene expression. Gene body methylation in honeybees likely improves the fidelity of gene expression by allowing transcription to initiate only at the promoter and not at intergenic regions. Gene body DNA methylation in plants, for instance, has been shown to suppress intragenic transcriptional start sites and anti-sense transcription, presumably by preventing transcriptional activation proteins from binding to the gene body and inappropriately activating transcription from cryptic promoters (Zhang et al., 2006).

Originally, it was reported that most DNA methylation occurs primarily in CpG sequences in honeybees (Lyko et al., 2010; Zemach et al., 2010; Chen et al., 2011). However, we have shown, by analyzing our own data, and by reanalyzing the data from Lyko et al. (2010), that there is actually more CHH DNA methylation in honeybees than CpG DNA methylation (Cingolani et al., 2013). The other laboratories that analyzed DNA methylation in the honeybee used software that removed most of the CHH DNA methylation, presumably because this type of DNA methylation occurs in less complex regions of the genome (i.e., CG poor) and are therefore harder to align to the reference genome. Also, multiple CHH methylation events in a single next-generation DNA sequencing (NGS) read are often, sometimes improperly, interpreted as poorly converted by bisulfite and thrown out. However, we validated that most of the CHH methylation events are real by alternative methods, such as sequencing honeybee genomic DNA after immunoprecipitation with anti-5mC antibodies, and enzymatic digestion of DNA at 5-hydroxymethylcytosine (5hmC) sites (Cingolani et al., 2013). We did confirm, however, like the other groups, that CpG DNA methylation is primarily in exons. Interestingly, we also found that CHH DNA methylation is primarily in introns, partly because introns are larger and have a lower CG content (Cingolani et al., 2013).

We were also the first group to find significant amounts of 5hmC in bees (Cingolani et al., 2013). 5hmC is an oxidized form of cytosine, and is presumably produced by the honeybee ortholog to the ten-eleven-translocation (TET) protein, a dioxygenase that converts 5mC to 5hmC, and is involved in epigenetic reprogramming in mammals (reviewed in Chia et al., 2011). Wojciechowski et al. (2014) recently confirmed the presence of 5hmC in honeybees and characterized the enzymatic function of the TET enzyme. Because of the uncertainty of whether 5hmC is a stable epigenetic mark, as some investigators believe (including us), or a transient DNA modification in the de-methylation pathway, as most investigators believe, we will not discuss 5hmC further in this review but will await future clarification on this topic.

**FIGURE 2:**
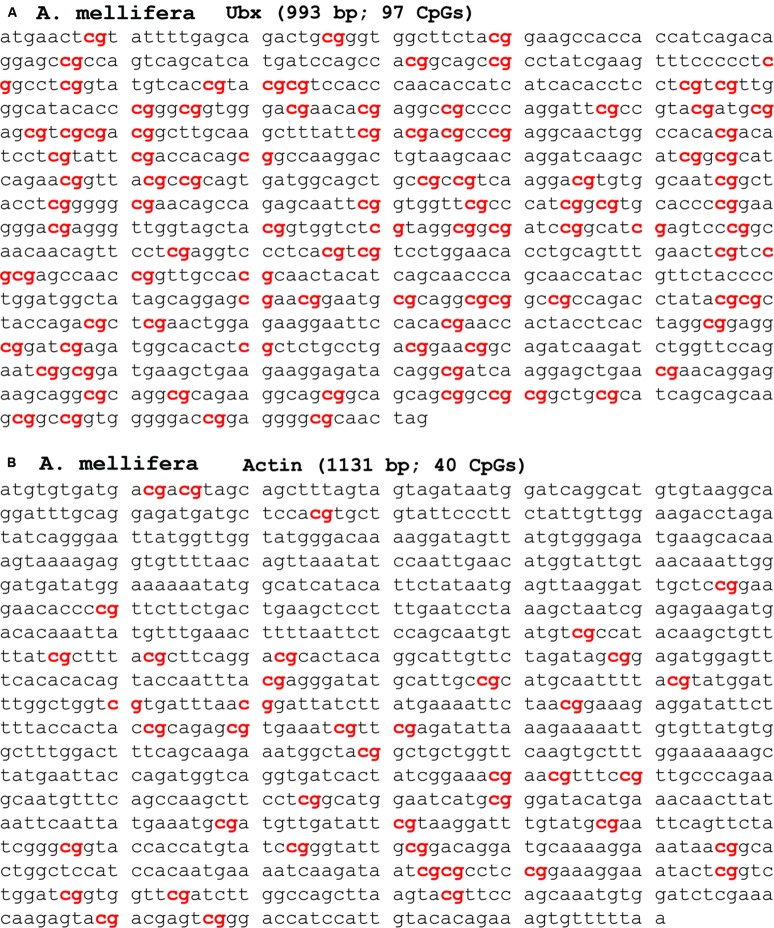
**Examples of high CG and low CG genes. (A)** *Apis mellifera* Ubx has 97 CG s in the coding region of a 993 base pair cDNA. The 5mC level of high CG -content genes, such as Ubx, is low. **(B)** *A. mellifera* actin has 40 CG s in the coding region of a 1131 base pair cDNA. The 5mC level of low CG -content genes, such as actin, is high.

Genome sequencing the honeybee showed that it has an unusual genome structure that we believe facilitates the generation of metastable epialleles (Elango et al., 2009). In honeybees, there are two types of genes based on CG content in exons (**Figure 1C**). Highly expressed, so-called housekeeping genes, which are expressed in all cells, have a lower CG content than low-expressed genes. This bimodal distribution of CG content in genes, which are called isobars, was first observed by a bioinformatics analysis of the newly sequenced honeybee genome (Jorgensen et al., 2007). The discovery of isobars in the honeybee genome was made prior to the mapping of the 5mC sites by whole-genome shotgun bisulfite sequencing by our laboratory and several other laboratories (Lyko et al., 2010; Zemach et al., 2010; Chen et al., 2011; Cingolani et al., 2013). Sodium bisulfite converts C to uracil (U) unless it is methylated (5mC), and whole genome shotgun bisulfite sequencing is used to map all of the 5mC sites in the genome (Xi and Li, 2009). Interestingly, all of the groups that performed whole-genome shotgun bisulfite sequencing to map the 5mC distribution in honeybees found, at first impression paradoxically, that the low-CG content genes have much more DNA methylation than the high-CG content genes (Lyko et al., 2010; Zemach et al., 2010; Chen et al., 2011; **Figure 1C**). We additionally found that CHH DNA methylation is also greater in the low-CG content genes than in the high-CG content genes. We proposed, since there is not a bimodal distribution of CHH sequences, that the same DNA methyltransferases (i.e., DNMT1 and DNMT3) methylate both CG and CHH sequences in a manner that is directly proportional to the level of gene expression (Cingolani et al., 2013). **Figure 2** shows an example of a high-CG content, low-5mC gene (Ubx, **Figure 2A**) and a low-CG content, high-5mC gene (Actin, **Figure 2B**). Both Ubx and Actin will be discussed as examples throughout this perspective.

To reiterate, low-CG content genes have more 5mC than high-CG content genes. This is counter-intuitive because it indicates that the greater the CG content, the less the DNA methylation, despite there being more cytosines (specifically, CpG sites) to methylate. However, high-CG content genes having low DNA methylation makes biological sense for the same reason that CpG islands (by definition, with high CG content) have low DNA methylation. The biological sense is based on the fact that 5mC has a much higher (up to 10-fold) mutation rate to thymidine (T) than non-methylated cytosine (Rakyan et al., 2001). Therefore, the more highly expressed genes would have more 5mC (**Figure 1B**), and, consequently, more of the cytosines would become thymidine. Consequently, in highly expressed genes, the CG-content would be expected to become lower-and-lower as more-and-more CGs are converted to TGs. The reason for the higher mutation rate of 5mC-to-T compared with C-to-T is that 5mC spontaneously deaminates at the 6-position to form T, which is a natural DNA base. However, unmethylated C deaminates to U, which is normally not present in DNA, and there are enzymes [specifically uracil *N*-glycosylase (UNG)] to remove the U bases in DNA (Rakyan et al., 2001). The diagrams in **Figures 1A,B** are a simplification for clarity purposes because the most highly expressed genes, which we will call “ultra-high,” usually have less DNA methylation than the medium and highly expressed genes in the gene bodies in both insects and mammals. This might be because the ultra-high expressed genes may have lost so many of their CpGs that there are not enough remaining to allow them to enter the most highly methylated class – in other words, the amount of DNA methylation that can occur in genes is saturated and peaks before it reaches equilibrium. The genetic code for certain amino acids and intra-exon RNA-splicing enhancers requiring CGs in their consensus sequencings are likely two additional reasons for retaining a few CGs in housekeeping genes.

As discussed in the next section, differential gene-body methylation might be a contributing factor to the emergence of eusociality. However, the bimodal distribution of CG content seems to be less of a contributor to eusociality than the bimodal distribution of DNA methylation. Bees, wasps, and ants all have bimodal distributions in DNA methylation in genes, but only bees and wasps have a bimodal distribution in CG content. In all three eusocial insects – bees, wasps and ants – the highly expressed genes are generally more methylated than the low-expressed genes (Sarda et al., 2012). Sarda et al. (2012) studied the evolution of gene-body DNA methylation in invertebrates and showed that silkworm (*Bombyx mori*), which has DNA methylation at appreciable levels in the genome, nevertheless does not have a bimodal peak of CG content in genes. This is similar to our finding of a unimodal peak of CHH sites but a bimodal peak of DNA methylation based on CG content, discussed earlier (Cingolani et al., 2013).

Interestingly, the silkworm has a bimodal peak in DNA methylation levels similar to the honeybee, in which highly expressed genes have higher levels of DNA methylation in the gene body. The unimodal peak in CG content but bimodal peak in DNA methylation levels seen in the silkworm genome also occurs in all ant species studied so far (Glastad et al., 2011; Bonasio et al., 2012; Bonasio, 2015). The bimodal peak in CG content in genes is not unique for the honeybee, however, because it is also seen in other invertebrates such as the sea anemone (*Nematostella vectensis*) and the sea squirt (*Ciona intestinalis*; Sarda et al., 2012). We conclude that while bimodal peaks in CG content and DNA methylation might facilitate the formation of metastable epialleles, they are not essential for the generation of metastabile epialleles. In the next section, we explore the possibility that metastable epiallele hyper-mutability, a key component of the EDGE hypothesis, is an emergent property of bimodal levels of DNA methylation in eusocial insects.

## METASTABLE EPIALLELE HYPER-MUTABILITY MIGHT BE AN EMERGENT PROPERTY OF BIMODAL LEVELS OF DNA METHYLATION

The movement from low-level rules to higher level sophistication is what we call emergence (Johnson, 2001).

The above quote is from Johnson’s (2001) best-selling 2001 book, “Emergence: the connected lives of ants, brains, cities, and software.” In the book, Johnson (2001) describes how a simple behavior, such as an increasing number of ants following a weak-and-winding scent trail laid down by one ant to a food supply, can lead to a complex behavior, such as all of the ants following a direct path to the food. Eusocial insects show many other examples of bottom-up behavior where workers follow simple rules that emerge into complex hive behaviors (Johnson, 2001). However, in contrast to human societies, there is little if any top–down behaviors in eusocial insects. For example, as mentioned above, the queen is best characterized as the “reproductive organ” in the hive and does little to influence the behaviors of the worker sub-castes (Johnson, 2001), who themselves follow simple rules that are programmed into their genomes and epigenomes. We believe that the differential methylation of genes based on the level of gene expression is just such a simple rule that can lead to complex emergent phenomena, such as metastable epiallele hypermutability and, ultimately, eusociality.

We hypothesize that an emergent property of low-expressed genes having low levels of DNA methylation is that they become more susceptible to epigenetic control, for the simple fact that they have more unmethylated cytosines. Highly expressed genes with high levels of DNA methylation can also potentially become metastable epialleles, but this would require differential de-methylation, such as by TET enzymes, in the germline cells after a stress response. In another review we presented a model for how oxidative stress can alter the function of the TET enzyme (Chia et al., 2011). However, what is the normal function(s) of gene body DNA methylation? In addition to preventing intragenic and antisense transcription within genes, mentioned above, one process that we and others have shown evidence to be regulated by gene body DNA methylation is alternative mRNA processing. For example, DNA methylation of cassette exons, at both CpG and CHH sites, correlates with their preferential exclusion in the mature mRNA (Lyko et al., 2010; Cingolani et al., 2013; **Figure 1D**). Furthermore, Li-Byarlay et al. (2013) have shown that RNA interference (RNAi) knockdown of DNMT3a, the *de novo* DNA methlyltransferase, alters RNA splicing and causes intron retention in hundreds of genes in the honeybee fat bodies. How DNA methylation affects alternative mRNA splicing is not known in bees, but in mammals, DNA methylation inhibits the binding of the transcription factor CCCTC binding factor (CTCF), which affects alternative splicing (Shukla et al., 2011). We speculate that there might be some biophysical processes involved too, since methylated DNA has a higher melting temperature (T_m_) than unmethylated DNA (Severin et al., 2011). Therefore, the increased T_m_ of methylated DNA might alter RNA polymerase translocation rates, cause pausing, and thereby affect the alternative mRNA splicing pattern.

One interesting observation is that most insects, such as honeybees, have relatively large amounts of DNA methylation (but much less than mammals), but *Drosophila* has very little DNA methylation (Lyko et al., 2000; Lyko, 2001). The reason for the scarcity in DNA methylation in *Drosophila* is that *Drosophila* appears to have lost Dnmt1, the maintenance DNA methyltransferase, which methylates hemizygous DNA after replication, and Dnmt3, the *de novo* DNA methyltransferase, which methylates unmethylated DNA. The existence of DNA methylation in *Drosophila* is controversial because the only cytosine methyltransferase orthologs in *Drosophila* is a homolog to DNA methyltransferase 2 (MT2), but this enzyme was shown to methylate transfer-RNA-Asp (tRNA_Asp_) and presumably not DNA (Goll et al., 2006). However, the controversy appears to be resolved (at least to some in the field) by a recent paper that shows CHH methylation, albeit at very low levels, in *Drosophila* in a manner that is independent of MT2 (Capuano et al., 2014). The authors were able to detect low levels of 5mC in *Drosophila* embryos in a two-step protocol of first immunoprecipitation of DNA with anti-5mC antibodies, followed by bisulfite sequencing of the immunoprecipitated DNA fragments (Capuano et al., 2014). Our laboratory has similar evidence for low levels of 5mC in *Drosophila* and we speculate that it is generated non-enzymatically by spontaneous methylation of cytosines by intrinsic alkylation of DNA.

We speculate that Dipterans (flies) and Coleopterans (beetles) lost DNA methyltransferases 1 and 3 because DNA methylation is redundant with histone modifications, such as H3K9me3 and H3K27me3, in repressing gene expression. Furthermore, we speculate that methylated DNA slows down DNA replication because of the higher melting temperature (T_m_) of methylated DNA compared with unmethylated DNA (Severin et al., 2011), which we mentioned earlier in the discussion of mRNA splicing. The predicted slowing down of DNA replication by DNA methylation is important in *Drosophila* because the first 10 syncytial nuclear divisions in the blastoderm embryo are in “hyper-drive” and are less than 8–10 min in duration (a world record, to our knowledge). Therefore, any process that slows down these rapid divisions would presumably be selected against because the faster-developing siblings would breed sooner (Ruden and Jackle, 1995).

## EPIGENETIC DIRECTED GENETIC ERRORS AND THE EVOLUTION OF CASTS IN HONEYBEES

Macroevolution requires selection of existing genetic variation to generate new species with greater fitness, but how does the sub-caste worker specialization increase when the effective population size of eusocial insects is so low (i.e., only one reproductive female per hive)? We mentioned group selection and kin-selection models at the beginning of this review, but they remain controversial in light of Dawkins’s (1976) “selfish gene” hypothesis. Dawkins (1976) argued that “selfish genes” that benefit the immediate survival and propagation of the “vessel” (the organism) would have much greater (and more immediate) selective advantage than altruistic genes that benefited the group. We speculate again, as we did in several other reviews, that one possible mechanism to facilitate genetic variation in the evolution of species is what we call the EDGE hypothesis (Ruden, 2005; Ruden et al., 2005a, 2008; Ruden and Lu, 2008).

**FIGURE 3:**
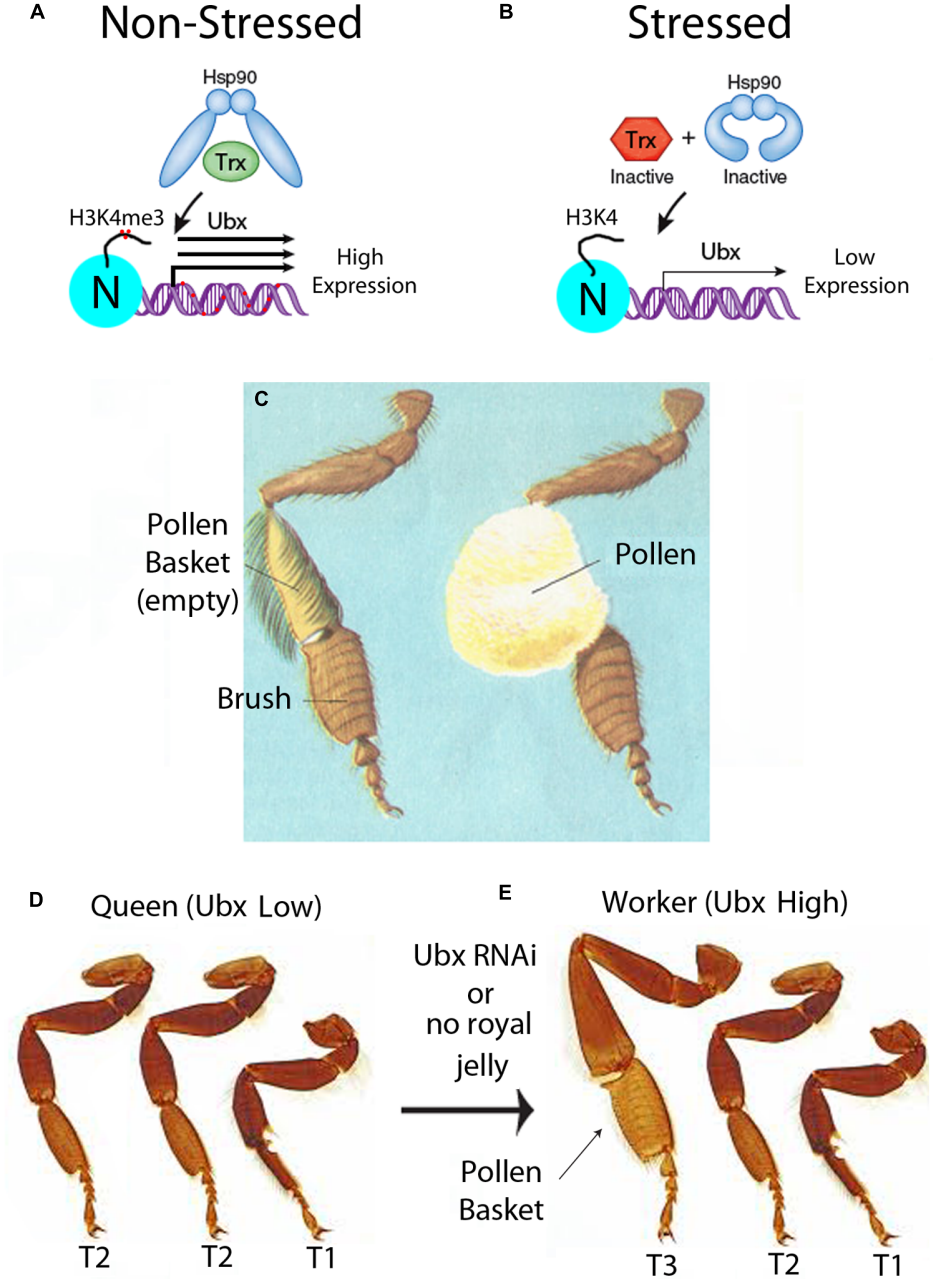
**Epigenetic control of development of the pollen basket in worker bees. (A)** In unstressed conditions, Hsp90 is functional and activates Trithorax (Trx), through the chaperone activity of Hsp90. The Trx group (TrxG) proteins tri-methylate histone 3 lysine 4 (H3K4me3) on the promoter nucleosomes (N) (red dots), and increase expression of Hox genes such as Ubx. Transcriptional activation of Ubx in bees increases the DNA methylation of the gene body (red dots), as shown in **Figure 1B**. In the epigenetic directed error hypothesis (EDGE), the repair of base substitutions caused by methylated cytosines increases the mutation frequency of not only of the methylated cytosine but also neighboring bases. This could lead to an increase in the mutation frequency of genes with metastable epialleles, such as in the Ubx gene. This figure is modified from a previous review from our laboratory and we retain the copyright (Ruden, 2011). **(B)** In stressed conditions, Hsp90 is inactive and cannot activate Trx, and transcription of Ubx is low. **(C)** Diagram of an empty and full pollen basket in forager bees. This diagram is used with permission from the Encyclopedia of Science, Copyright © The Worlds of David Darling (http://www.daviddarling.info/). **(D)** In queens, the pollen basket does not form because Ubx is low in T3. This causes an anterior transformation of the third thorax (T3) leg to look like the T2 leg. **(E)** In workers, the pollen basket forms because Ubx expression is high in T3. This figure represents a simplified representation of the homotic transformation that occurs when Ubx levels are reduced and are not meant to be accurate illustrations. This photograph is used with permission from Spike Walker, Wellcome Images, London (http://wellcomelibrary.org/).

In the simplest version of the EDGE hypothesis, the first step is the intra-caste selection of metastable epialleles that increase the specialization of a worker. The metastable epialleles could initially be generated by a stressful (i.e., non-optimal) environment, which would lead to a functional inactivation of Hsp90 (Rutherford et al., 2007a), which is a chaperone for many chromatin remodeling proteins (Ruden and Lu, 2008), including the Trithorax (Trx) protein (Tariq et al., 2009; **Figures 3A,B**). Hsp90 has been called a “capacitor for morphological evolution” because many previously cryptic phenotypes are revealed when stress inactivates Hsp90 protein and this alters multiple signaling pathways (Rutherford and Lindquist, 1998; McLaren, 1999; Rutherford and Henikoff, 2003; Rutherford et al., 2007a,b). The Trx protein, since it is a client for Hsp90, is an environmentally sensitive component of the Trx Group (TrxG) complex of proteins that is involved in maintaining transcriptional memory (i.e., activation) of the Hox genes, such as the Ultrabithorax (Ubx) gene during early embryogenesis in insects (Orlando et al., 1998). One of the enzymatic functions of the TrxG complex is trimethylation of histone 3 at lysine 4 (H3K4me3), which is an activating mark for transcription (Jenuwein and Allis, 2001).

**FIGURE 4:**
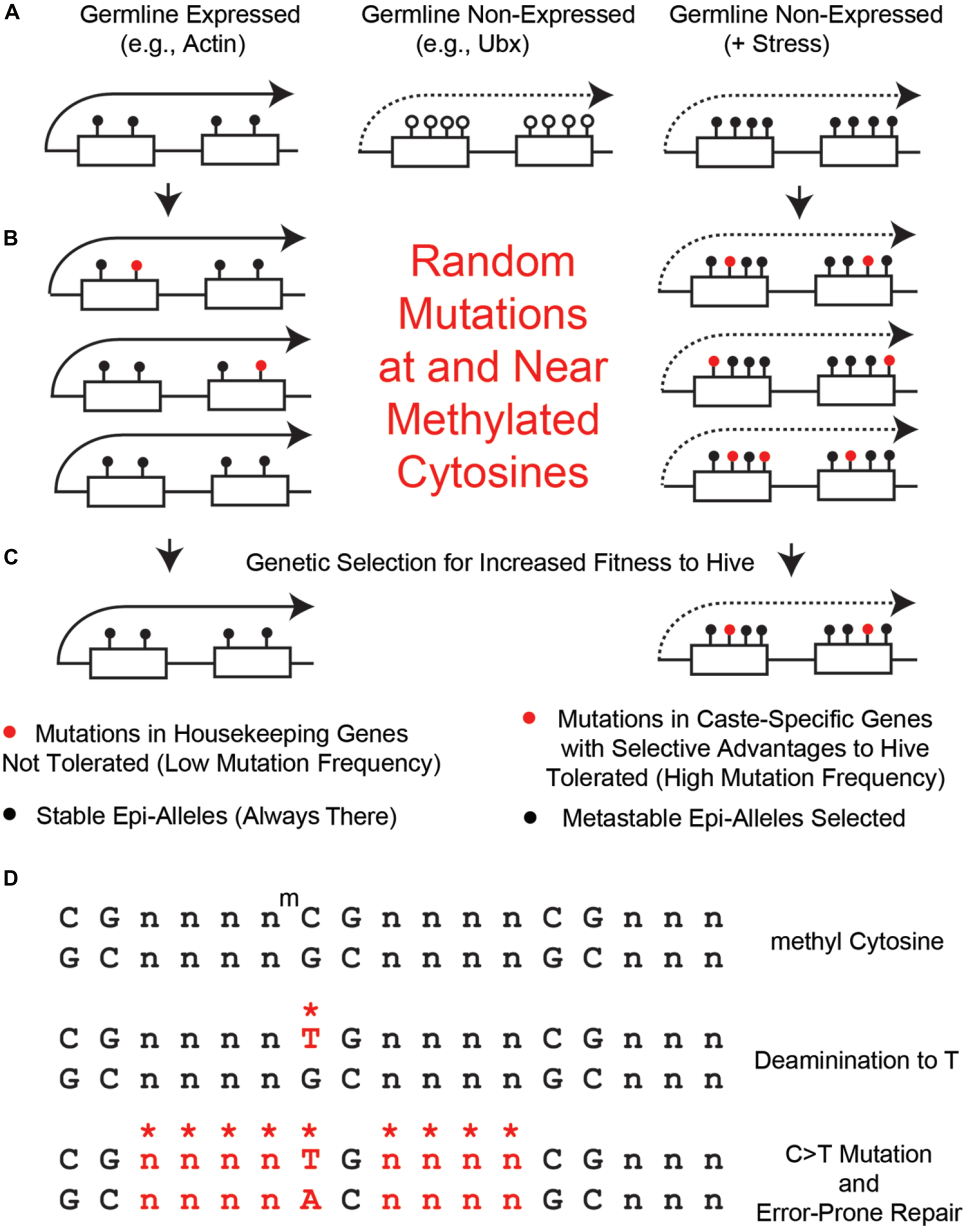
**Random mutations generated at and near methylated cytosines. (A)** Left, in germline high-expressed genes, such as actin, there are few CGs and high levels of 5mCs (black circles). Middle, in germline non-expressed genes, such as Ubx, there are many CGs and low levels of 5mCs (open circles). Right, in non-expressed genes in the presence of stress (+Stress), we propose that many of the CG s become methylated by Trx-switching-to-PCG, described in **Figure 2A**. **(B)** Left, few 5mCs in the few CG s in high-expressed genes become mutated to TGs (red circles). Right, few 5mCs in the many CG s in poor-expressed stressed genes become mutated to TGs. **(C)** Left, mutations in high expressed genes get removed by purifying selection. Right, mutations in metastable epialleles remain because of low levels of purifying selection. **(D)** Mutations in bases near CG s (*n) can be generated by error prone DNA repair of 5mC > TG mutations.

When Hsp90 is inactivated in a stressful environment, Hox genes such as Ubx would have lower expression, presumably because there would be less H4K4me3 histone marks at the promoters. Since stress inactivates the Trx protein (**Figure 3B**), then stress would be expected to cause an increase in the DNA methylation status of the Ubx gene. The reason for this is that, in the absence of the Trx protein, the gene would no longer be in an activated state but switch to a repressed state by the Polycomb Group (PcG) repressor proteins (Paro et al., 1998). It is not known whether this occurs in bees, but in mammals genes that are initially repressed by PcG proteins are often further repressed by intragenic DNA methylation during cellular differentiation (Deaton et al., 2011). We speculate that Ubx would have originally become a metastable epiallele in the proto-queen, who still has pollen baskets, because full pollen-baskets could immobilize her, and hence stress her, in the confines of the hive. In the EDGE hypothesis, the repair of base substitutions caused by methylated cytosines increases the mutation frequency of not only of the methylated cytosine, as mentioned above (Rakyan et al., 2001), but also neighboring bases because of error-prone DNA repair mechanisms (Ruden, 2005; Ruden et al., 2005a,b). This error-prone DNA repair could lead to an increase in the mutation frequency of genes with metastable epialleles, such as in the Ubx gene (**Figure 4D**). Through this “mutation-spreading” effect, the metastable epialleles could cause not only an increase in the mutation frequency of the exons, but also regulatory sequences in the adjacent promoters and introns. In other words, simply by becoming a metastable epiallele, the EDGE hypothesis predicts that the mutation frequency of a gene would increase. Fortuitously, genes with increased mutation frequencies are precisely those that need to be mutated to stabilize the metastable epialleles in a genetic manner.

How EDGE mutations generated in sterile workers are transmitted to the next generation in honeybees is a major issue that warrants discussion. One possible mechanism for transmitting the EGDE mutations to the next generation could be through honeybee workers who develop ovaries and become fertile after queen removal, as mentioned above (Feldmeyer et al., 2014). They could then directly transfer the mutations (as well as the metastable epialleles) to their offspring. Those mutations that are beneficial to the hive by stabilizing the metastable epialleles would have a selective advantage for the whole hive and would thereby be selected by group selection. An important consideration is that fertile-workers only have drone progeny (i.e., haploid males) and queens have both drone and worker progeny. This would necessitate that the metastable epialleles be transmitted through the male germline in the offspring of fertile workers. However, it is not clear whether worker-to-fertile-female conversions are frequent enough to explain the evolution of sterile-worker specializations.

Another possible mechanism for the transfer of EGDE mutations to the next generation is that that the queen can transmit EDGE mutations to her offspring directly, without having to go through a worker-to-fertile-female conversion process. Metastable epialleles have to be in either the queen or the worker (by definition), but affect them in different manners. Therefore, the genes that become metastable epialleles would be predicted to have a higher mutation rate by the EDGE process. Some of these mutations would not affect the queen (and therefore reproduction), but might stabilize the metastable epialleles that affect the workers. Support for the EDGE hypothesis is the fact that queen-specific genes mutate faster than worker-specific genes (Hunt et al., 2010; Helantera and Uller, 2014) This makes sense since queens breed much more frequently than fertile workers, which, as mentioned above, only occur when the queen is removed from the colony (Feldmeyer et al., 2014). Queens would therefore have a greater opportunity to transmit both metastable epialleles, and mutations in the metastable epialleles, to the offspring, than the fertile workers.

## EPIGENETIC DIRECTED GENETIC ERRORS AND THE EVOLUTION OF POLLEN BASKETS IN HONEYBEE WORKERS

A recent paper that we believe supports the EDGE hypothesis for intra-caste evolution in honeybees discusses the dimorphism in pollen basket formation in genetically similar queens and workers. This fascinating paper shows that the Hox gene Ubx, mentioned throughout this perspective, promotes pollen basket formation on the tibia of the hind legs in bees in the third thoracic segment (T3) (Medved et al., 2014). The pollen basket is a hollow indentation on the large and mostly bristle-free tibia segment that the forager bees use to store and transport impressive amounts of pollen (**Figure 3C**). In the queen, who does not collect pollen, the tibia is covered with hairs that would otherwise inhibit pollen collection. The investigators showed that reduction of Ubx levels in the workers by injecting inhibitory RNA (RNAi) into the worker embryos caused the hind legs to resemble that of the queens and become reduced in bristles (Medved et al., 2014). In *Drosophila*, mutations in Ubx, combined with other mutations in the bithorax complex (BXC), produced the famous four-winged fly that won Edward Lewis the 1995 Nobel Prize in Physiology and Medicine (Crow and Bender, 2004). Normally, *Drosophila* one pair of wings on the second thoracic segment (T2) and one pair of halteres (balancer organs that counteract the wing movement) on the third thoracic segment (T3). Lewis (1978) explained the Ubx phenotype as causing an anterior homeotic transformation of T3 to T2, hence, the famous four-winged fly. Ubx has a conserved 60 amino acid homeobox (Hox) domain, which is nearly identical from *Drosophila* to humans, and a highly variable transcriptional regulatory domain. Hox genes, such as Ubx, not only regulate segmentation during embryogenesis, but they also affect subtle changes in limb, brain, and other organ development.

Medved et al. (2014) found that mutations in the Hox gene, Ubx, causes complex fate decisions in each segment of the honeybee T3 legs. A simplification of the results of the Ubx-RNAi experiments in honeybees is that the third leg has a partial homeotic transformation to the second leg by a similar T3-to-T2 homeotic transformation as seen in *Ubx*-mutant flies (**Figures 3D,E**). As mentioned earlier, the way the nurse honeybee controls pollen basket development in workers is by withholding royal jelly. In honeybees, the targets of Ubx are not known, but the authors speculated on what might be occurring in honeybees, based on what is known in the much better characterized *D. melanogaster* genetic system. In honeybee queens, when they are fed royal jelly as larvae, the HDACi activity in the royal jelly could possibly help in the activation of expression of the likely Ubx-target genes, such as grunge (gug) and Ataxin-2 (Atx2), which play a role in the formation of bristles in *Drosophila* (Erkner et al., 2002; Al-Ramahi et al., 2007). Consequently, the authors speculate, this might be one reason why the T3 tibia segments in queens have bristles in the area of the pollen basket, while workers do not (Medved et al., 2014).

## EPIGENETIC DIRECTED GENETIC ERRORS IN NON-CG DINCULEOTIDES IN METASTABLE EPIALLELES

In the EDGE hypothesis, we propose that methylated cytosines are mutagenic not only in the 5mC sites but also in the surrounding bases. The reason we propose this broader-range of mutagenicity is because error-prone DNA repair mechanisms can increase the mutation frequency of surrounding bases while repairing 5mC > T base substitution mutations. Metastable epialleles, which have variable levels of 5mC, can occur in both somatic cells and germline cells, but they are generally referred to as simply “differentiated cells” when they occur in somatic cells. When metastable epialleles occur in somatic cells, they cannot be transmitted to the progeny. However, when a metastable epiallele occurs in a germline cell, then it can be transmitted to the progeny, as we and others have demonstrated in *Drosophila* (Sollars et al., 2003; Tariq et al., 2009).

It is not yet known whether there is a bimodal distribution of 5mC in bee germline cells, but for the sake of argument, let’s assume for this perspective that it is similar to what occurs in somatic cells – i.e., housekeeping genes have low CG-content and high levels of 5mC and low-expressed genes have high CG-content and low levels of 5mC (**Figure 4A**). Therefore, housekeeping genes, such as Actin (**Figure 4A**, left) would never be metastable epialleles because their few CG s are always heavily methylated – i.e., there can be no differential 5mC if it is always high. In contrast, low-expressed genes, such as Ubx, which is presumably not expressed at all in germline cells, would have high CG content but very little 5mC (**Figure 4A**, middle). We hypothesize that maternal stress can increase the DNA methylation in low-expressed genes, such as Ubx, and turn them into metastable epialleles (**Figure 4A**, right). This has not yet been demonstrated in any organism, but it should be possible to test this hypotheses in the laboratory once single-cell epigenomics techniques are further optimized (Teles et al., 2014).

In housekeeping genes, such as Actin, there would still be expected to be an increase in mutations near and surrounding the 5mC sites. However, since there is a great deal of purifying selection in housekeeping genes, that would make any deleterious mutations in such important structural genes selected against (**Figures 4B,C**, left). Also, the 5mC rate in housekeeping genes is so high that there has probably been a maximum change in CG-to-TG sequences so that no further such mutations can occur without having deleterious structural or regulatory changes to the gene. In contrast, in low-expressed genes, such as Ubx, there would be mutations in CG-sites that do not undergo as much purifying selection (**Figures 4B,C**, right). As mentioned above, while the Ubx Hox domain is a 60 amino acid sequence that is almost absolutely conserved from *Drosophila* to humans (Scott, 1986), the remaining amino acids, such as in the transcriptional regulatory domains, are amongst the most variable sequences in proteins (Ruden et al., 1991; Ruden, 1992).

In the Ubx-mutagenesis hypothesis for basket formation in honeybees, several questions arose during review of this manuscript. First, “How did Ubx changed its biological role without affecting fitness?” Second, “Is it possible that Ubx regulates both body plan and caste differentiation in honeybee but not in solitary insects?” Third, “Could some other gene(s) compensate for the supposed “functional loss” of Ubx in honeybee?” To answer these questions, we do not believe that the mutations in Ubx would necessarily affect fitness by causing a “functional loss.” Rather, we believe that the mutations in Ubx were most likely regulatory mutations in the promoter and introns and they represent a functional gain rather than a functional loss. Developmental genes have large and complex regulatory regions, such as individual enhancers for each of the eight stripes in segmentation genes such as fushi tarazu (Ohtsuki et al., 1998). The Hox genes in *Drosophila*, such as Ubx and Antennapedia (Antp), have enhancer regions 10s or even 100s of kilobases from the promoter regions (Calhoun et al., 2002; Calhoun and Levine, 2003). The Ubx gene in *Drosophila* has a complex array of alternative spliced products and an unusual mechanism for splicing the 74 kb intron that involves multiple steps of re-splicing the intron (Hatton et al., 1998). This re-splicing mechanism avoids competition between distant splice sites and allows removal of the 74 kb intron as a series of smaller RNA fragments (Hatton et al., 1998). The diverse array of transcriptional and RNA splicing regulatory sequences should allow Ubx to evolve multiple additional roles in caste formation without the need for other genes to compensate for it proposed “functional loss.”

## CONCLUSION

We propose an intra-caste model of evolution that is based on selection of metastable epialleles in worker bees that runs parallel to the macro-evolution and group selection of DNA mutations. Like the mythical world of Jaynus, the evolution of the most-fit sub-caste members occurs through the selection of metastable epialleles by group selection. However, our EDGE hypothesis expands upon the limited world of Jaynus, in which all of the organisms have exactly the same sequence, by proposing a mechanism to direct mutations to the metastable epialleles that were selected. These directed mutations can, in turn, stabilize and increase the penetrance of the metastable epialleles in future generations of superorganism colonies. Waddington, who is often considered the father of epigenetics, proposed a mechanism similar to the EDGE hypothesis in Waddington (1942) for the inheritance of acquired characteristics that were induced by stress. In follow-up experiments, in response to Waddington, we provide a possible epigenetic mechanism for how stress can reveal previously cryptic phenotypic information by the inactivation of Hsp90 (Ruden et al., 2003; Sollars et al., 2003). Finally, our EDGE hypothesis presented here provides a possible mechanism for the stabilization of metastable epialleles, thereby allowing the evolution of castes and sub-castes in eusocial insects.

## Conflict of Interest Statement

The authors declare that the research was conducted in the absence of any commercial or financial relationships that could be construed as a potential conflict of interest.
